# From Metal Thiobenzoates to Metal Sulfide Nanocrystals: An Experimental and Theoretical Investigation

**DOI:** 10.3390/nano2020113

**Published:** 2012-04-03

**Authors:** Zhihua Zhang, Wen Pei Lim, Chiong Teck Wong, Hairuo Xu, Fenfang Yin, Wee Shong Chin

**Affiliations:** Department of Chemistry, National University of Singapore, 3 Science Drive 3, Singapore 117543, Singapore; Email: zhihua.zhang@ap.rhodia.com (Z.Z.); peipeigy@yahoo.com.sg (W.P.L.); wongct@ihpc.a-star.edu.sg (C.T.W.); chmxhr@nus.edu.sg (H.X.); fenfang.yin@ap.rhodia.com (F.Y.)

**Keywords:** metal sulfide nanoparticles, thiobenzoates, silver sulfide, copper sulfide, indium sulfide, cadmium sulfide

## Abstract

A simple preparation of metal sulfide nanoparticles via the decomposition of thiobenzoate precursors at room temperature is presented and discussed. Long chain alkylamines were found to mediate the breakdown of metal thiobenzoates, such as those containing Ag, Cu, In and Cd, to produce uniform Ag_2_S, Cu_2−x_S, In_2_S_3_ and CdS nanoparticles respectively. The long chain amines are assumed to play dual roles as the nucleophilic reagent and the capping agent. It was found that sizes of the nanoparticles can be controlled by changing the type of amine used, as well as the molar ratio between amine and the precursor. We performed DFT calculations on a proposed mechanism involving an initial nucleophilic addition of amine molecule onto the thiocarboxylates. The proposed reaction was also confirmed through the analysis of by-products via infrared spectroscopy. On the basis of this understanding, we propose to manipulate the stability of the precursors by coordination with suitable stabilizing groups, such that the reaction kinetics can be modified to generate different nanostructures of interest.

## 1. Introduction

The synthesis of metal sulfide nanocrystals has attracted much interest for both fundamental research and technological applications in the past decades [1,2,3,4,5,6]. Metal sulfide nanocrystals have been prepared by a wide range of synthetic methods, one of which involves the direct decomposition of molecular precursors [7,8,9,10,11,12,13,14]. Molecular precursor approach has recently been developed as an efficient route to prepare monodispersed semiconductor nanocrystals [7,10,15] and, in some cases, unique shape-control has been achieved [9,11,16]. 

One of the earliest precursors used for preparing metal sulfides in the literature is *N*,*N*’-dialkyl dithiocarbamate. In this preparation, the precursor was injected into hot coordination solvents such as trioctylphosphine oxide (TOPO) under nitrogen atmosphere at high temperatures [7,8,9,10,11]. Metal bis(benzylthiolates) [12] and metal salts of alkylxanthate [17,18,19] have also been used as precursors to prepare nanocrystalline sulfides of zinc, cadmium or lead via pyrolysis at 150–400 °C. It was found that Lewis base such as hexydecylamine (HDA), trioctylphosphine (TOP) or tributylphosphine (TBP) could lower the reaction temperature for the alkylxanthate precursors. Recently, there are also several reports on the preparation of ternary and metal sulfide nanocrystals via the decomposition of thiocarboxylate precursors [20,21,22,23,24]. Most of these syntheses, nevertheless, require elevated or refluxing temperatures.

In this paper, we report a generalized precursor method operating at room temperature for the synthesis of various metal sulfide nanocrystals. The precursors we use are metal thiobenzoates [M_x_(SCOC_6_H_5_)_y_ or simply MTB]. These MTB precursors are air-stable and could be readily prepared from thiobenzoic acid and the corresponding metal salts following the known literature method [25]. We illustrate the generality of this MTB method by preparing four types of semiconductors from both transition and main group metals: Ag_2_S, Cu_2−x_S, In_2_S_3_ and CdS. We discuss in this report the basis of our approach and also attempt to understand the reaction mechanism through theoretical Density Functional Theory (DFT) calculations. With this insightful knowledge, we demonstrate how to manipulate the stability of the precursor, and thus the reaction kinetics, to generate different nanostructures.

## 2. Experimental Section

### 2.1. Synthesis of Precursors and Metal Sulfide Nanocrystals

Commercially available compounds such as thiobenzoic acid (Fluka), ether, ethanol, chloroform (all from J. T. Baker), sodium bicarbonate (Dumont), silver nitrate (Merck), indium chloride (Fluka), acetonitrile, cadmium acetate, copper chloride, 2,2’-bipyridine, octylamine, dodecylamine and oleylamine (all from Aldrich) were used as received.

All of the MTB precursors used in this paper (M = Ag, Cu, In, Cd; TB = thiobenzoate) were prepared according to literature methods [25]. The products obtained were washed with ethanol, dried, and recrystallized from chloroform or ether. Purity of the crystals was checked with microanalysis and their decomposition profiles were investigated by thermogravimetric analysis (TGA). 

For the synthesis of silver sulfide nanoparticles, AgTB (3 mmol) was stirred in toluene (5 mL) at room temperature, and then added with 1.8 mmol of an amine. A homogeneous clear brown solution formed quickly and the solution was stirred for a further 3 hours. After that, 10 mL of ethanol was added to induce turbidity in the mixture. The brown or dark brown nanoparticles are isolated by centrifugation, washed several times with ethanol and acetone, and then dried under vacuum. The resulting powder can be easily re-dispersed in toluene, hexane, chloroform and other non-polar solvents.

The experimental procedure is similar for the synthesis of copper sulfide nanoparticles, except that the reaction mixture turned blue and was stirred overnight. Dark brown or black copper sulfide nanoparticles were isolated, which can be re-dispersed in non-polar solvents such as toluene, hexane and chloroform. 

The synthesis of cadmium sulfide nanoparticles is similar to silver sulfide and copper sulfide, except that the reaction mixture turned yellow and was stirred for 4 hours at room temperature. Pale yellow product was separated by centrifugation after adding 10 mL of ethanol. The product isolated, after washing with ethanol and acetone, can be re-dispersed in chloroform, hexane and toluene.

For the synthesis of indium sulfide nanoparticles, InTB (0.3 mmol) was stirred in toluene (5 mL) at room temperature, and then 1.2 mmol of octylamine (OA) was injected to give a yellow solution. After stirring for 6 hours, 10 mL of ethanol was added to induce the formation of turbidity. The particles were purified similarly to the previous procedure. For the preparation of oleylamine-capped In_2_S_3_ nanoparticles, it was found necessary to further add 40 µL of propylamine to speed up the reaction.

### 2.2. Characterizations

TGA was recorded on a SDT 2960 Simultaneous DTA-TGA by heating approximately 10 mg of the precursor under inert N_2_ flow (flow rate 90 mL/min) at a heating rate of 10 °C/min. X-ray diffraction (XRD) analysis was carried out on a Siemens D5005 X-ray powder diffractometer with Cu Kα radiation (40 kV, 40 mA). The powdered sample was mounted on a sample holder and scanned with a step size of 2θ = 0.05° in the range of 20° to 90°. UV-Visible spectra were recorded using a Shimadzu UV-2550 UV/Vis spectrophotometer. FT-IR spectra were recorded using a FTS 165 Bio-Rad FTIR spectrophotometer in the range of 4000–400 cm^−1^ on KBr or nujol mulls. Transmission electron microscopy (TEM) images were obtained on a 100 kV JEM-100CXII TEM and 200 kV JEOL 2010F microscope. Samples were prepared by placing a drop of the dispersed nanoparticles onto a copper grid with carbon film, and were allowed to dry in a desiccator.

### 2.3. Computational Methodology

Calculations were performed using the hybrid density functional B3LYP [26,27] method with the effective core potential LanL2DZ [28,29,30,31] basis set. All reported energies include zero point energy corrections. All calculations were performed using the Gaussian 98 [32] suite of programs. Optimization was performed without any constraints and the optimized structures were verified to be equilibrium structures or transition states from frequency calculations. An equilibrium structure is characterized by all real frequencies while a transition state has one and only one imaginary frequency.

## 3. Results and Discussion

### 3.1. Thermal Behavior of the MTB Precursors

**Figure 1:**
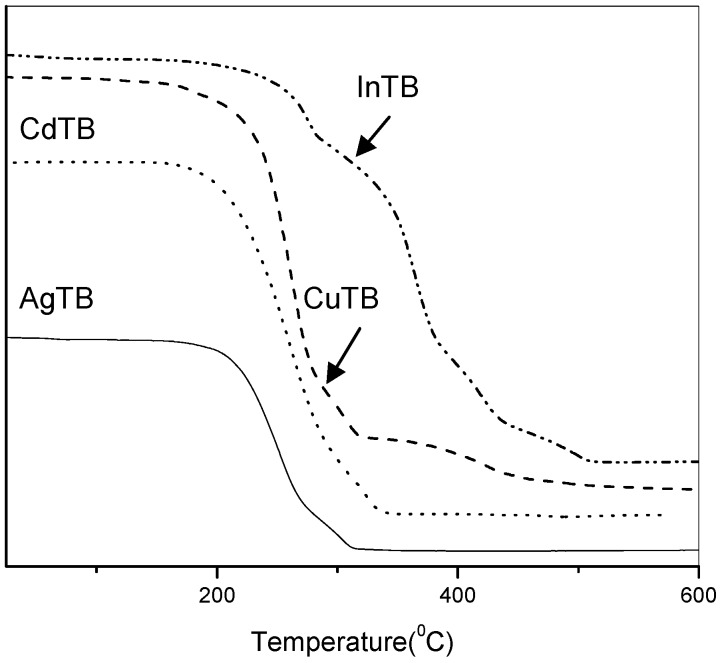
TGA profiles of the various MTB precursors. Detailed decomposition results are tabulated in Table 1.

The decomposition profiles of the four MTB precursors were investigated and their TGA curves are presented in Figure 1 and Table 1. The analysis indicated a clear onset of decomposition at ~ 200–300 °C in each case. The residual weight after each complete decomposition was found to be close to the expected remaining weight of the corresponding metal sulfide. The slightly higher measured values are possibly due to the presence of some nonvolatile carbon-containing side-products [33].

**Table 1:** Decomposition onset and weight of residue obtained from TGA plots in Figure 1.

Precursors	Decomposition onset (°C)	Weight of residue (%)	
		Measured	Expected for MS_x_
**AgSCOC_6_H_5_**	172	51.4	50.6
**CuSCOC_6_H_5_**	155	36.5	35.4
**In(SCOC_6_H_5_)_3_**	196	33.6	31.0
**Cd(SCOC_6_H_5_)_2_**	163	38.5	35.9

The decomposition pathway is expected to be similar to typical thiocarboxylate compounds as proposed by Hampden-Smith [33]:

M (SCOC_6_H_5_)_x_ → MS_x/2_ + x/2 (C_6_H_5_CO)_2_S   (1) 

Hence, in solvents such as carboxylic acid and TOPO, these complexes were found to decompose to the corresponding metal sulfides at elevated temperatures. In our studies, nevertheless, we have found that the MTB precursors decompose readily at room temperature by reacting with various alkylamines. In Section 3.2 below, we first characterize the sizes and morphologies of the nanoparticles prepared. We then present the DFT results on our proposed mechanism in Section 3.3.

### 3.2. Preparation and Characterization of Metal Sulfide Nanoparticles

#### 3.2.1. Silver Sulfide Nanoparticles

**Figure 2:**
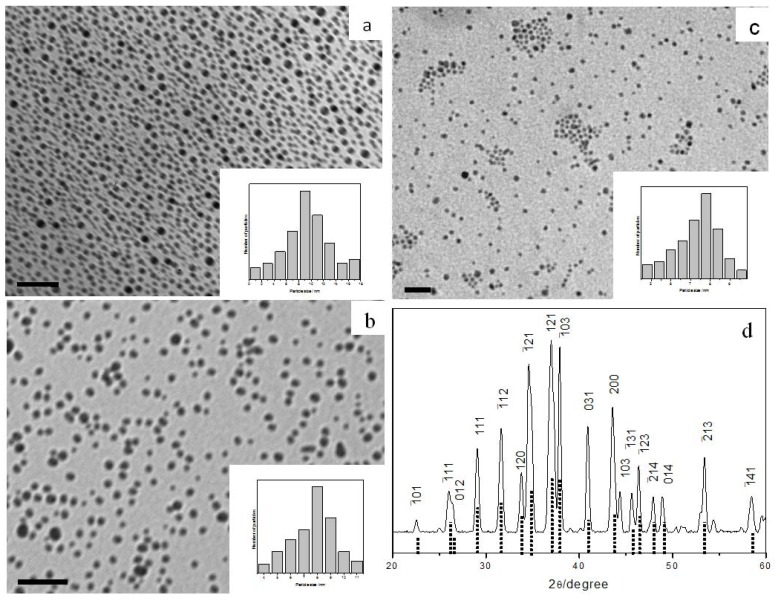
TEM images and size histograms of Ag_2_S nanoparticles prepared with different amines: (**a**) octylamine, (**b**) dodecylamine, and (**c**) oleylamine; all scale bar = 50 nm. (**d**) XRD pattern of the monoclinic Ag_2_S nanoparticles prepared. The standard pattern of α-phase Ag_2_S (JCPDS 14-72) is shown as dotted lines below the pattern.

Ag_2_S is a semiconductor with a narrow band gap of 1.08 eV [34]. When AgTB precursor was stirred with octylamine (OA) in toluene at room temperature, monodispersed Ag_2_S nanocrystals can be isolated after 3 hours (Figure 2a). Typical XRD pattern of the nanoparticles prepared from AgTB is shown in Figure 2d. The diffraction pattern revealed good monoclinic crystallinity and fitted well with the α-phase of bulk Ag_2_S (JCPDS 14-72). This is known to be the stable silver sulfide phase which commonly exists at room temperature.

The same α-phase silver sulfide nanocrystals were obtained when the reaction was carried out using other amines such as dodecylamine (DDA) and oleylamine (OLA). TEM analysis showed that spherical nanoparticles with reasonable size distribution were produced at room temperature in all these cases. The Ag_2_S nanocrystals obtained from different amines, however, are slightly different in sizes as shown by the size histograms in Figure 2a–c. Thus, average particle diameters of 9.2 ± 1.9, 8.3 ± 1.5 and 7.5 ± 0.9 nm are obtained for reaction with OA, DDA and OLA respectively. In conclusion, the Ag_2_S particle sizes can be controlled by varying the chain length of the alkylamine used, and longer-chain amines tend to produce smaller-sized particles.

**Figure 3:**
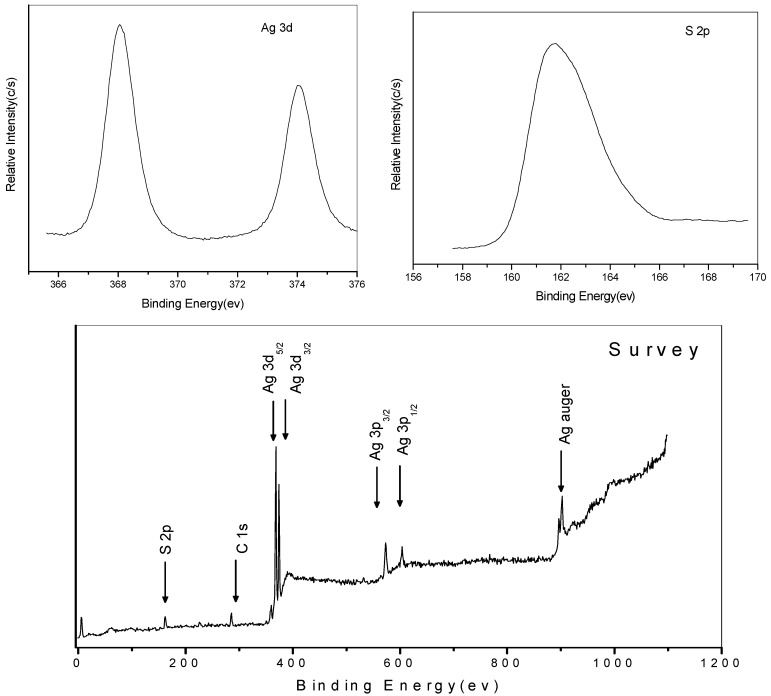
(**Bottom**) XPS survey scan, (**Top**, Left to Right) Ag 3d and S 2p elemental photoelectron peaks of nanocrystalline Ag_2_S.

Typical XPS spectra of the Ag_2_S nanocrystals are shown in Figure 3, with binding energies corrected with reference to the C 1s peak at 284.7 eV. The doublet arising from Ag 3d_5/2_ and 3d_3/2_ was detected at 368.0 and 374.1 eV respectively, while the S 2p photoelectron peak appears at 161.7 eV. These values are close to those of bulk Ag_2_S [35]. There is no O 1s peak (531.0 eV) detected on the spectrum, indicating that there is no by-product such as Ag_2_SO_4_ (368.3 eV) or Ag_2_O (368.4 eV) produced. Peak area analysis of the Ag 3d_5/2_ and S 2p peaks, after accounting for elemental sensitivity factors, gives an elemental ratio of 1.97:1 for Ag to S.

#### 3.2.2. Copper Sulfide Nanoparticles

**Figure 4:**
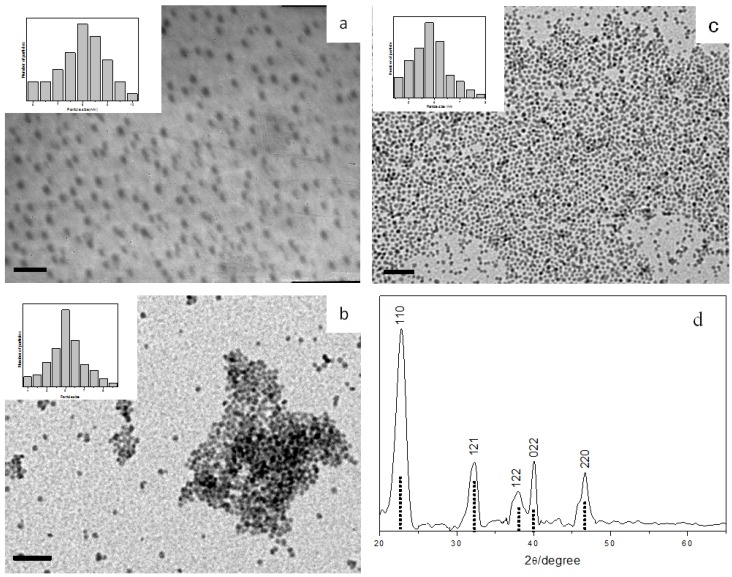
TEM images and size histograms of Cu_2−x_S nanoparticles prepared with different amines: (**a**) octylamine (OA), (**b**) dioctylamine (DOA), and (**c**) oleylamine (OLA); all scale bar = 50 nm. (**d**) XRD pattern of the rhombohedral Cu_2−x_S nanoparticles prepared. The standard pattern of JCPDS 85-1693 Cu_2−x_S phase is shown as dotted lines.

Similarly, we found that CuTB precursors readily decompose by reacting with alkylamines at room temperature to give uniform Cu_2−x_S nanoparticles. TEM analysis (Figure 4a–c) indicated average diameters of 8.1 ± 1.1, 6.1 ± 0.5 and 5.8 ± 0.4 nm for the nanoparticles produced from OA, dioctylamine (DOA) and OLA, respectively.

It is well known that copper sulfides exist in many different phases and compositions (Cu_x_S, x: 1 → 2). Non-stoichiometric copper sulfide is readily formed and has been utilized as superionic conductor or p-type semiconductor. XPS peak area analysis gave an elemental Cu to S ratio of 1.72:1 for our samples, thus corresponding to x ~0.28 in the Cu_2−x_S general formula. The XRD pattern in Figure 4d clearly revealed the rhombohedral structure, which fits well with the standard bulk Cu_2−x_S phase (JCPDS 85-1693). 

While DOA also reacts with CuTB to produce copper sulfide nanoparticles at room temperature, we notice that the growth rate is slightly slower and the particles produced are smaller than those prepared with primary amine (*i.e*., OA). We believe that, since OA is less bulky compared to DOA, it attacks the precursor with less hindrance and thus causes the reaction to occur at a faster rate. In addition, DOA is expected to play a better role to efficiently prevent the nanoparticles from aggregation and thus will tend to produce smaller particles. On the other hand, our experiments confirmed that tri-substituted amine does not result in the formation of Cu_2−x_S, probably due to its bulkiness.

#### 3.2.3. Indium Sulfide Nanoparticles

β-In_2_S_3_ is an n-type semiconductor with a band gap of 2.0–2.2 eV [36]. It has promising applications in the preparation of green and red phosphors for photoconductors and photovoltaics [37,38,39]. In addition, it can serve as a host for a number of metal ions to form semiconducting and/or magnetic materials. In_2_S_3_ nanocrystals have not received as much attention so far, due to the lack of a straightforward preparation methodology. In this case, we have successfully prepared these nanoparticles by decomposing InTB precursor in alkylamines. 

**Figure 5:**
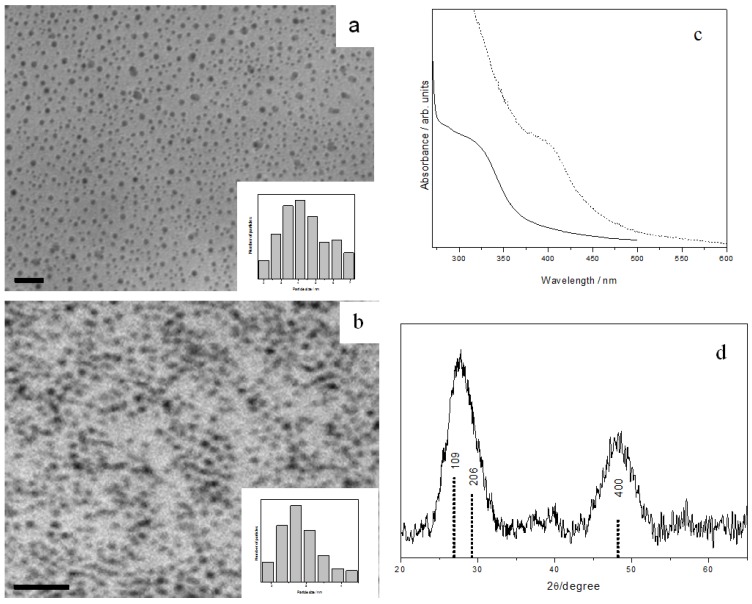
TEM images and size histograms of In_2_S_3_ nanoparticles prepared with different amines: (**a**) OA, and (**b**) OLA + trace amount of propylamine, all scale bar = 20 nm. (**c**) UV-Vis absorption spectra of OA-capped (dashed line) and OLA-capped (solid line) In_2_S_3_ nanoparticles. (**d**) XRD pattern of the In_2_S_3_ nanoparticles prepared. The standard JCPDS 32-0456 pattern of β-phase In_2_S_3_ is shown as dotted lines.

Reacting InTB with OA could produce spherical In_2_S_3_ with an average diameter of 3.7 ± 0.6 nm (Figure 5a). However, reaction of InTB with OLA or DOA could not proceed at room temperature until the addition of a trace amount of a short chain amine. Thus, upon adding propylamine into OLA, In_2_S_3_ nanoparticles with average diameter of 2.6 ± 0.4 nm could be isolated (Figure 5b). The In_2_S_3_ nanoparticles prepared from OLA and OA show a UV-Vis absorption onset at 355 nm (3.50 eV) and 437 nm (2.84 eV), respectively (Figure 5c). Compared to the band-gap of bulk In_2_S_3_ (2.2 eV or 564 nm) [36], it is clear that the excitonic transition is blue-shifted due to strong quantum confinement in these In_2_S_3_ nanocrystals. Figure 5d shows the XRD pattern of the as-prepared In_2_S_3_ nanoparticles, confirming the tetragonal β-phase of In_2_S_3_ (JCPDS 32-0456).

#### 3.2.4. Cadmium Sulfide Nanoparticles

**Figure 6:**
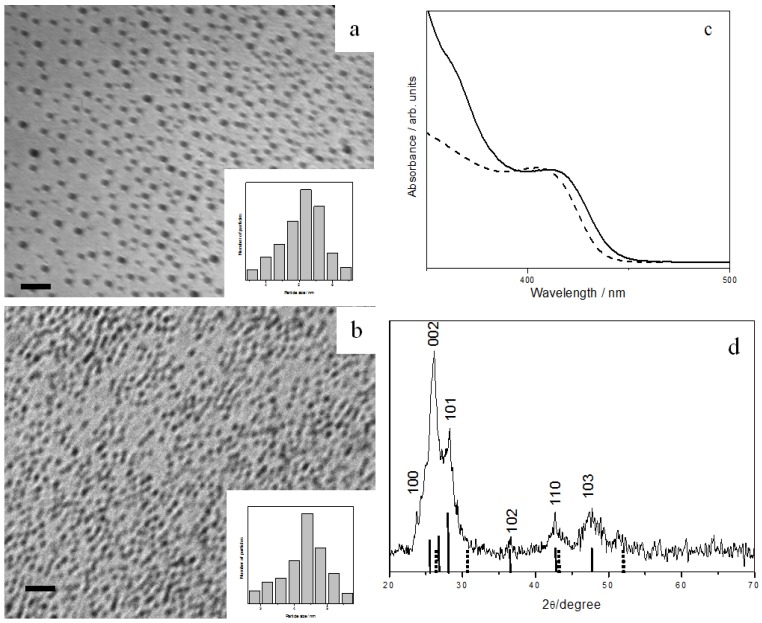
TEM images and size histograms of CdS nanoparticles prepared with different amines: (**a**) OA, and (**b**) OLA + a trace amount of propylamine, all scale bar = 20 nm. (**c**) UV-Vis absorption spectra of OA-capped (solid line) and OLA-capped CdS nanoparticles (dashed line). (**d**) XRD pattern of OA-capped CdS nanoparticles with wurtzite structures. The standard patterns of CdS phases are shown as sticks below the diffractogram: wurtzite (solid line) and zinc blende (dashed line).

CdS is one of the most studied metal sulfides, due to its various applications. When CdTB precursor was mixed with OA, spherical CdS nanoparticles with an average diameter of 5.3 ± 0.7 nm were produced (Figure 6a). No reaction had happened, however, in the sole presence of OLA at room temperature. Again, CdS nanoparticles with an average diameter of 4.4 ± 0.3 nm were obtained upon adding a small amount of propylamine into the OLA (Figure 6b).

The monodispersity of the prepared CdS nanoparticles is manifested in their absorption spectra, which exhibit a clear distinct band rather than a shoulder or threshold (Figure 6c). The band-edge absorption is blue-shifted, occurring at 432 nm for OA-capped and 424 nm for OLA-capped nanocrystals. These gave an estimated particle diameter of 4.9 and 4.0 nm respectively from the Brus equation, in good agreement with those obtained from TEM analysis. CdS is known to exist in two structures: Cubic zinc blende phase and hexagonal wurtzite phase. In our sample, XRD analysis (Figure 6d) suggested a wurtzite crystal structure (JCPDS 41-1049). This observation is interesting, since CdS nanoparticles with zinc blende structure are often produced in ambient conditions, whereas wurtzite structure is often obtained at high temperatures.

### 3.3. Proposed Mechanism for the Formation of Nanoparticles from the MTB Precursors

#### 3.3.1. The Role of Amine in the Reaction

From the above experimental results, we have confirmed that long chain alkylamines such as OA and OLA can react with the thiobenzoate precursors at room temperature to produce monodispersed metal sulfide nanoparticles. Comparatively short chain amines such as propylamine and ethylamine yield larger particles that precipitate quickly from the reaction mixtures. By mixing a small amount of these shorter chain amines into the long chain amines, on the other hand, enables monodispersed nanoparticles to be isolated again. Thus, the long chain amines are needed in this reaction as capping groups to efficiently prevent aggregation of the nanoparticles. 

After repeating the reactions using different types of amine, we could also conclude that the types of amine used will affect the rate of formation of the specific nanocrystals. Thus, for OLA and aliphatic amines with chains longer than 14 carbon atoms, In_2_S_3_ nanoparticles cannot be produced at room temperature. Hence, heating was needed to initiate this particular reaction. On the other hand, the reaction can be induced at room temperature when a small amount of shorter chain amines are added into the long chain amine. 

An important aspect of our findings is that MTB precursors are broken down by amines at room temperature. We have found that in other common capping reagents, e.g., TOPO, thiols or carboxylic acids, the decomposition of MTB precursors can only occur at elevated temperatures. We determined the onsets of these reactions by slowly heating up the MTB precursors in these media, and the results are summarized in Table 2. We could hence conclude that the decomposition of thiobenzoates in amines is not a simple pyrolysis process.

**Table 2:** Comparison of the decomposition onsets from TGA and reaction in different media for the various MTB precursors.

	AgTB	CuTB	InTB	CdTB
Decomposition onset (TGA)	172 °C	155 °C	196 °C	163 °C
OA	Room T	Room T	Room T	Room T
Octanethiol	~85 °C	~90 °C	~130 °C	~115 °C
TOPO	~130 °C	~130 °C	~160 °C	~150 °C
Octanoic acid	~145 °C	~140 °C	~170 °C	~155 °C

In addition to the above, we have also confirmed that tertiary amines such as trioctylamine do not react with the MTB precursors even at reflux conditions. Secondary amines, such as DOA, can produce monodispersed nanoparticles from CuTB and AgTB precursors at room temperature, but not with InTB and CdTB. Thus, it seems that amines with an active hydrogen atom are needed for the reaction to proceed at room temperature. 

**Scheme 1:**
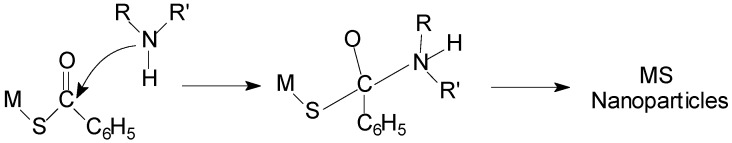
A generalized reaction scheme for the initial attack of alkylamine onto metal thiobenzoate.

On the basis of all the above observations, we propose a general reaction mechanism as follows. The reaction probably arises from an initial attack of the amine group onto the electron-deficient (*i.e*., electrophilic) carbonyl carbon of the MTB precursor [40], and this is followed by an elimination of an amide to produce the metal sulfides (Scheme 1):

For the InTB and CdTB precursor, the reaction with secondary DOA occurs only at elevated temperatures. We believe this is because the bulkier DOA could not access the carbonyl carbon of these di- and tri-substituted MTB precursors readily. As a comparison, we have also performed reactions with shorter chain secondary amines, such as diethylamine and dipropylamine. It was found that reactions of these secondary amines with MTB can occur readily at room temperature. The particles produced, however, are rather large in size, as expected from our proposition that amines are playing dual roles as the nucleophilic reagent and the capping agent. From these results, it can be seen that properties such as coordination ability, steric hindrance, solubility, as well as stability constants are all important factors in the successful preparation of nano-sized crystals from the MTB precursors.

#### 3.3.2. Results of DFT Calculations

**Figure 7:**
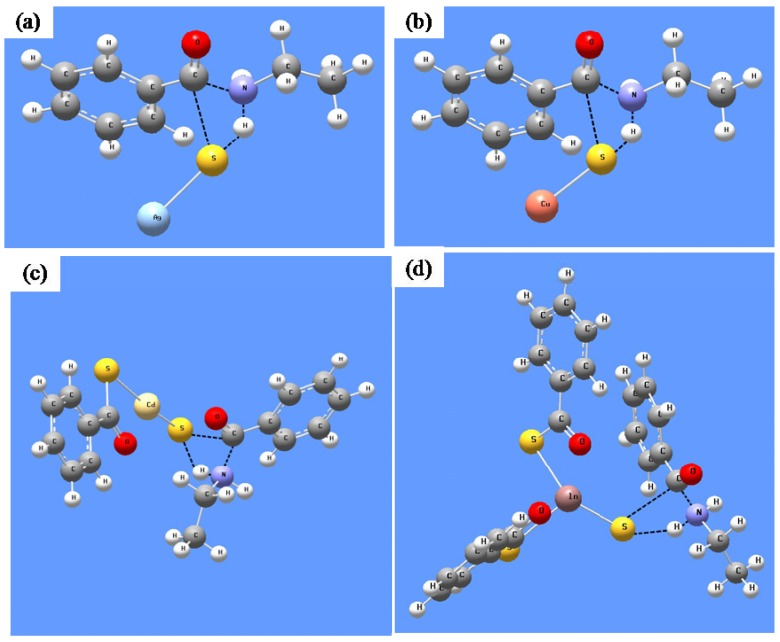
B3LYP/LanL2DZ optimized transition state structures for theethylamine-mediated decomposition of the respective MTB precursors.

In order to support the proposed mechanism in Scheme 1, we performed DFT calculations by modeling the reaction between MTB precursors with a simple amine-ethylamine. For all the four types of precursors, B3LYP/LanL2DZ optimized transition state structures were located as shown in Figure 7. The zero-point corrected enthalpy change values and energy barriers are tabulated in Table 3. For comparison, important geometric parameters of the optimized ground and transition state structures are presented in Table 4.

Thus, as can be seen in Figure 7, a transition state structure resembling the nucleophilic addition-elimination mechanism proposed is obtained for all the MTB precursors studied. Clearly, a C-N bond (~1.5–1.6 Å in Table 3) is formed between the nucleophilic amine and the electrophilic carbonyl carbon of MTB in the transition state. A simultaneous elimination of amide is also evident from the lengthening of one C-S bond from ~1.8 Å to ~2.7 Å. We notice also that one of the metal-sulfur bonds is shortened during this addition-elimination process towards the formation of metal-sulfur monomer. 

In Table 3, we noted that the DFT predicted activation energy values are similar, (~110 kJ mol^−1^) for all the four precursors studied. This low energy barrier is in agreement with our observation that these reactions can occur readily at room temperature. Moreover, all the reactions were predicted to give an exothermic enthalpy ranging from 4 to 33 kJ mol^−1^. The calculations further suggested that reactions of AgTB and CuTB will be fast, partly driven by the large exothermic enthalpy change.

**Table 3:** B3LYP/LanL2DZ predicted values of energy barrier and enthalpy change for the ethylamine-mediated decomposition of the respective MTB precursors.

Precursor	Energy barrier (kJ mol^−1^)	Enthalpy change (kJ mol^−1^)
Ag(SCOPh)	109.7	−33.3
Cu(SCOPh)	109.2	−27.3
Cd(SCOPh)_2_	108.0	−6.7
In(SCOPh)_3_	115.3	−4.0

**Table 4:** Comparison between the B3LYP/LanL2DZ optimized initial state and transition state structures for the ethylamine-mediated decomposition of the respective MTB precursors.

Precursor	Bond length in precursor (Å)		Bond length in transition state structures (Å)			
	M-S bond ^a^	C-S bond	M-S bond ^a^	C-S bond	C-N bond	N-H bond ^b^
Ag(SCOPh)	2.540	1.820	2.398	2.660	1.603	1.061
Cu(SCOPh)	2.348	1.812	2.173	2.669	1.601	1.060
Cd(SCOPh)_2_	2.632	1.803	2.454	2.801	1.564	1.102
In(SCOPh)_3_	2.725	1.777	2.373	2.608	1.628	1.035

^a ^Length of the shortened M-S bond; ^b ^N-H bond length of ethylamine = 1.017 Å.

In order to further elucidate the role of different amines, we also performed B3LYP/LanL2DZ calculations for reactions mediated by different amines. Using a AgTB precursor as the example, transition state structures similar to that shown in Figure 7a were obtained and the calculation results are summarized in Table 5. Similarly, the formation of a new C-N bond, lengthening of the C-S bond and shortening of the Ag-S bond are predicted. 

It is important to highlight that DFT calculations predicted the bond dissociation energy of AgS-COPh as 266.0 kJ mol^−1^, while that of Ag-SCOPh was predicted as 206.4 kJ mol^−1^. Thus, our studies suggest that the stronger S-C bond is broken instead of the weaker Ag-S bond in the presence of amine. In Table 5, variations between different amines was predicted to be rather similar, except that the energy barrier is slightly smaller and the exothermic enthalpy is larger in the case of OA and had, as compared to DOA. All these DFT results provided good support to our experimental observations and the proposed mechanism.

**Table 5:** B3LYP/LanL2DZ calculated results for the various amine-mediated decomposition of AgTB.

Amine ^a^	N-H bond length (Å)	Energy barrier (kJ mol^−1^)	Enthalpy (kJ mol^−1^)	Bond length in transition state structures (Å)			
				Ag-S bond ^b^	C-S bond ^b^	C-N bond	N-H bond
EA	1.017	109.7	−33.3	2.398	2.660	1.603	1.061
OA	1.017	107.6	−35.1	2.399	2.670	1.600	1.061
HDA	1.017	107.0	−36.2	2.398	2.680	1.599	1.061
DOA	1.019	108.8	−22.1	2.393	2.945	1.573	1.060

^a ^EA = Ethylamine, OA = Octylamine, HDA = Hexadecylamine, DOA = Dioctylamine;^ b ^Bond lengths in AgTB precursor: Ag-S = 2.540 Å, C-S = 1.820 Å.

#### 3.3.3. FT-IR Analysis of the Reaction Mixtures

**Figure 8:**
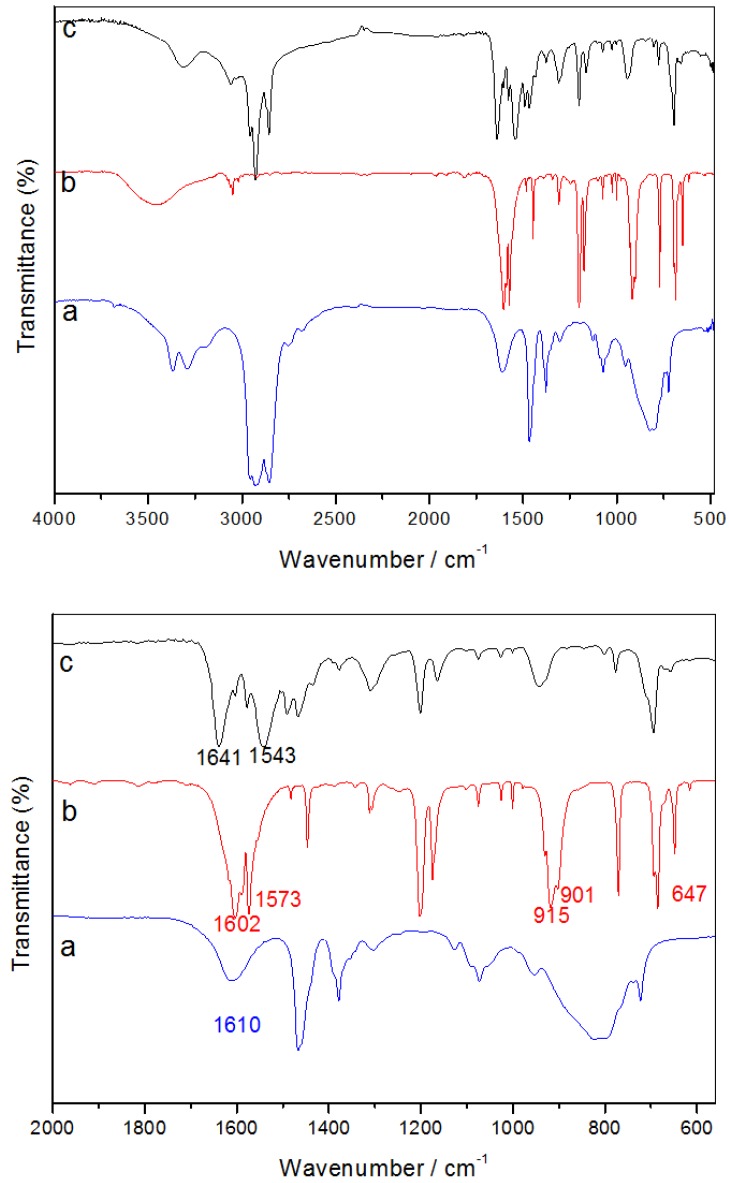
Full (top) and zoom-in (bottom) FTIR spectra of (**a**) OA, (**b**) AgTB precursor, and (**c**) the reaction mixture of AgTB with OA.

In order to further support the proposed mechanism, we monitored the reaction at room temperature using FTIR analysis. Reaction mixtures of the respective MTB precursor with OA were concentrated through rotary evaporation and their FTIR spectra were measured. Figure 8 shows a typical FTIR spectrum of such reaction mixture, together with the spectra of pure OA and the precursor for comparison. 

The FTIR spectrum of pure AgTB shows two strong bands at 1602 and 1573 cm^−1^, arising from C=O stretching. These are slightly lower in frequency as compared to υ(C=O) in thiobenzoic acid, due to its strong coordination to the metal ions [41]. Lower frequency peaks at 915, 901 and 647 cm^−1^ resulting from the C-S bending vibration and O-C-S deformation were also observed in the pure AgTB spectrum. On the other hand, the FTIR spectrum of pure OA shows a pair of fairly strong asymmetric and symmetric stretching of primary N-H bands at 3376 and 3292 cm^−1^, and also another N-H bending mode at 1610 cm^−1^.

In comparison, the reaction mixture AgTB + OA gives a IR peak of the C=O group at 1641 cm^−1^ (Table 6). This position is characteristic of the −C=O bond stretching in amides [41]. Besides, secondary amides often exhibit a relatively strong bending band at about 1543 cm^−1^, attributed to a combination of a C-N stretching band and an N-H bending band. Meanwhile, the doublet of N-H is replaced by a single sharp band at 3310 cm^−1^, typical of the N-H stretching frequency of a secondary amine or an amide. Clearly, the N-H bending mode, existing at 1610 cm^−1^ in pure OA, has disappeared in the reaction mixture.

**Table 6:** A comparison of IR spectral bands of AgTB, OA and the mixture of AgTB + OA.

Sample	Peak Assignments * (cm^−1^)					
	υ_s_(N-H)	υ_s_(C-H)	δ_s_(N-H)	υ_s_(C=O)	υ_s_(C-S)	δ(O-C-S)
OA	3376	2923	1610	-	-	-
	3292	2861				
AgTB	-	3049	-	1602	915	647
				1573	901	
AgTB + OA	3310	2928	-	1641	-	-
		2853		1543		

* υ_s_: stretching; δ_s_: N-H bending; δ: deformation.

Thus, the above FTIR analysis confirms the attack of the OA molecule onto the carbonyl carbon of the AgTB precursors to form an intermediate secondary amide adduct. In addition, it was also found that the vibrational peaks of C-S bending and O-C-S deformation detected in AgTB had disappeared from the mixture. This further confirms the breaking down of these bonds as suggested by DFT calculations in Section 3.3.2. 

### 3.4. Stability of Precursor and Its Influence on the Formation of Nanoparticles

In summary, we described above a general precursor method that can be used to prepare metal sulfides at room temperature. Since the injection of reagent is carried out at ambient conditions, special handling techniques are thus not necessary during preparation. In addition, there is just one parameter to monitor once the type of amine is chosen, *i.e.*, the relative amine-to-precursor ratio. This particular simplicity has allowed us to advantageously adopt the method in the preparation of homogeneous nanoparticles/polymer composites, for example, for electrode applications [42].

With a good understanding of the reaction mechanism, we propose to modify the stability of the precursors for controllable growth and crystal engineering. It is known that the growth of colloidal nanocrystals is controlled by a balance between nucleation and growth processes [43,44]. A successful synthetic scheme should start with a burst of nucleation events followed by a controllable growth stage, without either prolonged nucleation or ripening. In addition, anisotropic growth is favored by a sustained high monomer concentration. Hence, as a rule of thumb, a good precursor should be stable enough to prevent individual nucleation to occur, while sufficiently easy to break down to ensure a steady supply of monomers. We illustrate below our attempt to modify the stability of a AgTB precursor to achieve controllable formation of nanoparticles.

**Figure 9:**
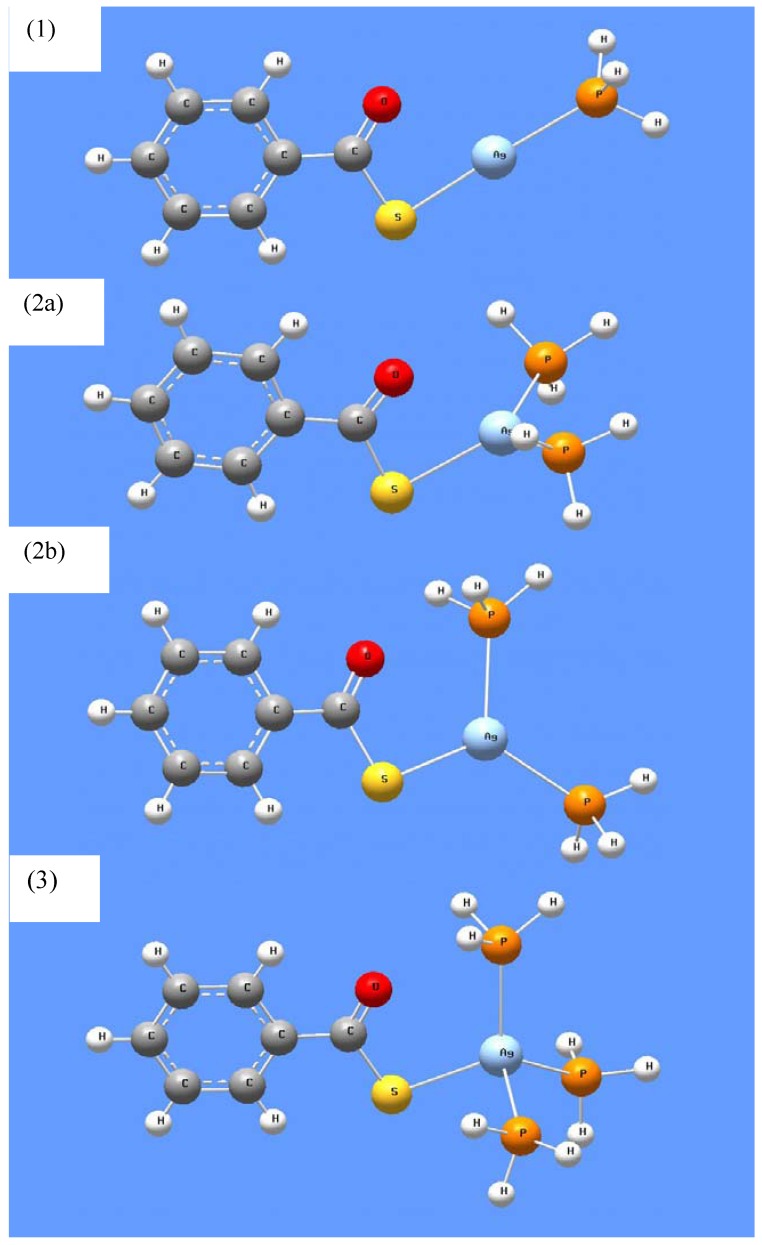
B3LYP/LanL2DZ optimized structures of PH_3_-coordinated AgTB complexes. The number of coordinated PH_3_ = 1, 2 and 3 in structure (**1**), (**2**) and (**3**) respectively.

By dissolving AgTB in trioctylphosphine (TOP) prior to the addition of amine, we found that heating to a temperature greater than 80 °C was required to produce Ag_2_S nanoparticles. In addition, we could obtain β-phase Ag_2_S as the kinetically-driven product by optimizing the reaction temperature and amine-to-precursor ratio [22]. Thus, TOP acts as the stabilizing reagent to AgTB in this case. We performed DFT calculations on complexes of AgTB coordinated to PH_3_ molecules (as a simpler analog of TOP) and the results are shown in Figure 9. In Table 7, DFT calculations predicted that three PH_3_ groups could be coordinated to AgTB, with an overall stabilization of ~109 kJ mol^−1^.

**Table 7:** B3LYP/LanL2DZ predicted effect of PH_3_ coordination to AgTB.

Structure ^a^	Complex	Energy + ZPE (Hartree ^b^)	Enthalpy stabilization (kJ mol^−1^)
-	AgTB	−500.73344	-
-	PH_3_	−8.24658	-
1	AgTB(PH_3_)	−509.01069	−80.5
2a	AgTB(PH_3_)_2_	−517.26284	−95.1
2b	AgTB(PH_3_)_2_	−517.26420	−98.7
3	AgTB(PH_3_)_3_	−525.51484	−109.4

^a^ The fully optimized structures are shown in Figure 9; ^b^ 1 Hartree = 2625.502 kJ mol^−1^.

**Figure 10:**
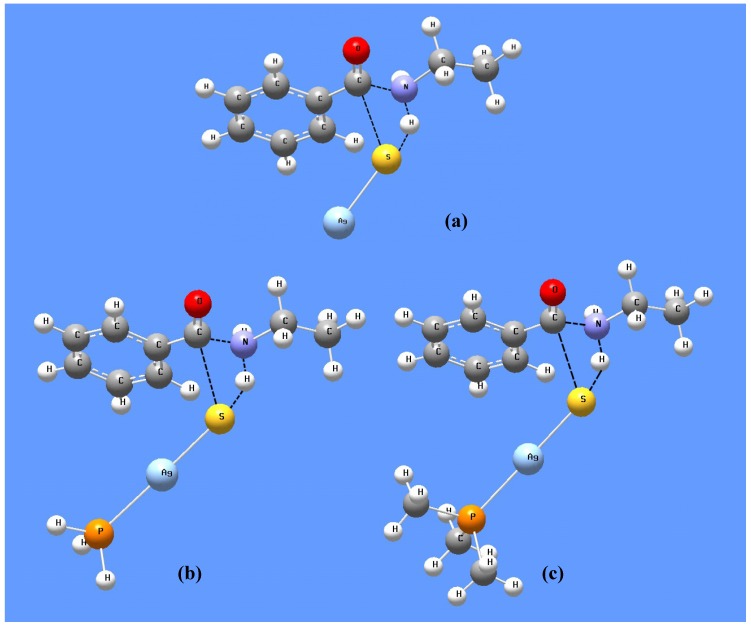
B3LYP/LanL2DZ optimized transition state structures for the reaction between C_2_H_5_NH_2_ and (**a**) AgTB; (**b**) AgTB(PH_3_); (**c**) AgTB(PMe_3_).

In addition, we have also located the transition states for the proposed amine-mediated nucleophilic attack onto the complexes by DFT calculations. We modeled the reaction using ethylamine attacking onto three different precursors: AgTB, AgTB(PH_3_) and AgTB(PMe_3_). The optimized transition state structures and their important geometrical parameters are compared in Figure 10 and Table 8. The DFT predicted energy barriers are 109.7 kJ mol^−1^, 124.1 kJ mol^−1^ and 128.6 kJ mol^−1^ for these three precursors respectively. The slightly higher barrier (~14–19 kJ mol^−1^) predicted for reactions involving AgTB(PH_3_) and AgTB(PMe_3_) is in agreement with our experimental observations.

Thus, we demonstrated that the desired control over nucleation and growth can be achieved through optimizing the delicate balance between the amount of activator (amine) and stabilizer (TOP). We have indeed found this to be general and applicable to most of the MTB precursors studied. For instance, we have varied the type of activator (from OA to dodecanethiol) and stabilizer (from TOP to tributylphosphite) to produce Cu_2−x_S of different crystalline phases [23]. By further changing the activator to difunctional amine such as ethylenediamine, dendritic Cu_2−x_S crystals were produced, giving well-defined snowflakes and blossom-like shapes [24].

**Table 8:** Important B3LYP/LanL2DZ geometrical parameters for the various transition state (T.S) structures, compared with those in the precursor complex (P) and ethylamine (EA).

Precursor complex (P)	Bond length (Å)						
	Ag-S bond		C-S bond		C-N bond	N-H bond	
	P	T.S	P	T.S	T.S	EA	T.S
AgTB	2.540	2.398	1.820	2.660	1.603	1.017	1.061
AgTB(PH_3_)	2.495	2.382	1.822	2.716	1.585		1.079
AgTB(PMe_3_)	2.496	2.390	1.822	2.731	1.579		1.087

## 4. Conclusions

In summary, we have reported here a simple route to prepare various types of metal sulfide nanoparticles readily from metal thiobenzoate precursors under ambient conditions. Such a simple and specific process may be utilized for the production of nanostructures and nanocomposites, and for applications such as nanofabrication and nanopatterning at ambient temperature.

Principles we have demonstrated in this report include: (1) by choosing a suitable capping agent that can initiate an attack onto the functionality of the precursor, a relatively less stable intermediate may be generated and this can lead to breaking down of the precursor at room temperature. We believe this also explains why precursors such as metal dithiocarbamate or metal alkylxanthate could react with amine to generate the corresponding semiconductors at temperatures well-below their decomposition onsets [12,15]; (2) by choosing a suitable coordination group, stability of the precursor may be optimized such that the decomposition can be moved to a kinetically-controlled regime. We believe this general principle can be used to guide the preparation of other types of metal chalcogenides from suitable precursors.
